# Spontaneous preterm birth and single nucleotide gene polymorphisms: a recent update

**DOI:** 10.1186/s12864-016-3089-0

**Published:** 2016-10-17

**Authors:** Ishfaq A. Sheikh, Ejaz Ahmad, Mohammad S. Jamal, Mohd Rehan, Mourad Assidi, Iftikhar A. Tayubi, Samera F. AlBasri, Osama S. Bajouh, Rola F. Turki, Adel M. Abuzenadah, Ghazi A. Damanhouri, Mohd A. Beg, Mohammed Al-Qahtani

**Affiliations:** 1 King Fahd Medical Research Center, King Abdulaziz University, PO Box 80216, Jeddah, 21589 Saudi Arabia; 2 Center of Excellence in Genomic Medicine Research, King Abdulaziz University, Jeddah, Saudi Arabia; 3 Faculty of Computing and Information Technology, King Abdulaziz University, Rabigh, Saudi Arabia; 4 Department of Obstetrics and Gynecology, Faculty of Medicine, King Abdulaziz University, Jeddah, Saudi Arabia; 5 KACST Innovation Center in Personalized Medicine, King Abdulaziz University, Jeddah, Saudi Arabia

**Keywords:** Preterm birth, Genes, Single nucleotide polymorphism (SNP)

## Abstract

**Background:**

Preterm birth (PTB), birth at <37 weeks of gestation, is a significant global public health problem. World-wide, about 15 million babies are born preterm each year resulting in more than a million deaths of children. Preterm neonates are more prone to problems and need intensive care hospitalization. Health issues may persist through early adulthood and even be carried on to the next generation. Majority (70 %) of PTBs are spontaneous with about a half without any apparent cause and the other half associated with a number of risk factors. Genetic factors are one of the significant risks for PTB. The focus of this review is on single nucleotide gene polymorphisms (SNPs) that are reported to be associated with PTB.

**Results:**

A comprehensive evaluation of studies on SNPs known to confer potential risk of PTB was done by performing a targeted PubMed search for the years 2007–2015 and systematically reviewing all relevant studies. Evaluation of 92 studies identified 119 candidate genes with SNPs that had potential association with PTB. The genes were associated with functions of a wide spectrum of tissue and cell types such as endocrine, tissue remodeling, vascular, metabolic, and immune and inflammatory systems.

**Conclusions:**

A number of potential functional candidate gene variants have been reported that predispose women for PTB. Understanding the complex genomic landscape of PTB needs high-throughput genome sequencing methods such as whole-exome sequencing and whole-genome sequencing approaches that will significantly enhance the understanding of PTB. Identification of high risk women, avoidance of possible risk factors, and provision of personalized health care are important to manage PTB.

**Electronic supplementary material:**

The online version of this article (doi:10.1186/s12864-016-3089-0) contains supplementary material, which is available to authorized users.

## Background

Preterm birth (PTB) is defined by the World Health Organization (WHO) as the birth of a baby before the completion of 37 weeks of gestation [1]. Global estimates for 2012 have revealed PTB to be one of the two top causes of child mortality second only to pneumonia and accounting for more than a million deaths each year [2, 3]. PTB is a worldwide significant clinical and public health problem. Data based on 2010 national estimates of 184 countries has shown 11 % (range 5–18 %) prevalence of PTB resulting in 15 million preterm babies [4]. Majority of PTBs (60 %) occur in Africa and South Asia, however, rich and industrially advanced countries also share the problem with a prevalence of about 6–7 %. Among the rich nations, the United States has the highest (10 %) prevalence. Recent data [4] for top 10 countries with highest number of PTBs (Table 1) and with highest rates of PTB per 100 live births (Table 2) are shown. In addition to the short term or immediate implications during the first month of neonatal life, PTB has long term lifelong consequences for the premature babies, their families, and for the overall population. Global Burden of Disease estimates show that PTB accounts for 3.1 % of all Disability Adjusted Life Years, more than that for HIV and malaria [5, 6]. Preterm neonates are many times more prone to problems including low blood sugar, jaundice, sepsis, pulmonary dysfunction, ophthalmological disorders, and long-term neurocognitive deficits [7, 8]. The frequent need for intensive care hospitalizations is associated with enormous emotional and financial burden. Estimates for the United States show that preterm-related death constitutes about 35 % of all infant deaths and preterm complications cost more than $26 billion (may even be $50 billion according to other estimates that include long term costs) to the U.S. health services [9]. Recent studies have found PTB infants to be at a long term disadvantage and even at increased risk of mortality during early adult years [10]. Not only were the PTB infants more likely to die in early childhood but also were more prone to mortality at 18–36 years of age due to cardiovascular, endocrine, and respiratory problems [10]. Even late preterm babies born at 34–36 weeks gestation were at higher risk compared to the term birth babies (37–42 weeks). The repercussions of PTB can even be felt in the subsequent generations as PTB individuals are less likely to reproduce [11]. Women who were born preterm have increased chances of having PTB thus continuing the vicious cycle of neonatal mortality and morbidity.

**Table 1:** Top 10 countries with greatest number of preterm birth (PTB) in 2010^a^

Name of the country	Number of PTBs (% of global total)	Preterm birth rate (% of livebirths in the country)
India	3519100 (23.6)	13.0
China	1172300 (7.8)	7.1
Nigeria	773600 (5.2)	12.2
Pakistan	748100 (5.0)	15.8
Indonesia	675700 (4.5)	15.5
The United States of America	517400 (3.5)	12.0
Bangladesh	424100 (2.8)	14.0
The Philippines	348900 (2.3)	14.9
The Democratic republic of Congo	341400 (2.3)	11.9
Brazil	279300 (1.9)	9.2

^a^Adapted from [4]

**Table 2:** The 10 countries with the highest rates of preterm birth (PTB) per 100 live births in 2010^a^

Name of the country	Number of PTB per 100 live births
Malawi	18.1
Comoros	16.7
Congo	16.7
Zimbabwe	16.6
Equatorial Guinea	16.5
Mozambique	16.4
Gabon	16.3
Pakistan	15.8
Indonesia	15.5
Mauritania	15.4

^a^Adapted from [4]

According to the severity of the shortened gestation, PTBs are subdivided into extremely preterm (less than 28 weeks) constituting about 5 % preterm births, severe or very preterm (28–31 weeks) constituting about 15 %, moderate preterm (32–33 weeks) constituting about 20 % while the majority (60 to 70 %) are late preterm or near term births (34–36 weeks) [4, 8]. A representative distribution of PTBs for singleton pregnancies in the United States for 2013 is presented in Table 3 (as an example of a country with complete birth record information). Of all the preterm births, about 70 % are spontaneous PTBs including 45 % that occur as a result of preterm labor and additional 25–30 % that occur as a result of preterm premature rupture of membranes (PPROM). The remaining about 30 % preterm births are due to an obstetrician’s decision to induce preterm delivery of the baby because of adverse and deteriorating uterine environment [8].

**Table 3:** Distribution of preterm births (PTB) for singleton pregnancies in the United States for the year 2013^a^

PTB Category	Gestation age (weeks)	Incidence in USA 2013	Percentage of PTB
Extremely preterm	<28	0.58	5.9
Severe or very preterm	28–31	0.96	9.9
Moderately preterm	32–33	1.18	12.2
Late preterm or near term	34–36	6.99	72.0

^a^Adapted from United States National Vital Statistics Reports 2015 [132]

In view of the importance and urgency of the problem of PTB, the Institute of Medicine (IOM) [9] under the aegis of the National Research Council of the United States convened the Committee on “Understanding Premature Birth and Assuring Healthy Outcomes” in 2007. The goals of the committee were to address the up-to-date status of science and clinical research of PTB, asses the health and financial impact of the problem of the PTB, and suggest a framework of recommendations to prevent and minimize the debilitating burden of the problem. The deliberations of the committee resulted in an exhaustive report on the PTB in 2007 [9] with up-to-date review of literature, assessment and recommendations. In addition to this exhaustive report there have been several reviews on the problem of PTB [2, 4, 12, 13] published recently. The number of studies on genetic polymorphism in relation to PTB has dramatically multiplied since the IOM report. However, the information about these genetic polymorphisms is dispersed and not available on a single platform. The purpose of the current review was to discuss and tabulate all recent studies on genetic polymorphisms associated with potential risk for PTB that have been published since the IOM report of 2007. A comprehensive literature search was done on preterm birth and SNPs in PubMed (www.ncbi.nlm.nih.gov/pubmed/). The terms used for searching the database were: preterm birth, PTB, premature birth, preterm premature rupture of membranes, PPROM, premature rupture of membranes, PROM, pregnancy outcome, single nucleotide polymorphism, SNP, gene variants, gene polymorphism, and genomics of preterm birth. The search was restricted to years 2007 to 2015 and search terms were used in combination to maximize the retrieval of relevant articles and for exclusion of unrelated literature. The reference lists of the included research articles were further scanned for identification of additional topic-relevant research articles. The initial search resulted in identification of about 300 journal articles. The titles of these articles were preliminarily scanned for relevance to the topic of preterm birth and genomics. Irrelevant articles, case reports, and editorials were excluded. Finally, 92 journal articles on association of preterm birth and single nucleotide polymorphisms were deemed relevant and included in this review.

### Risk factors associated with PTB

**Fig. 1:**
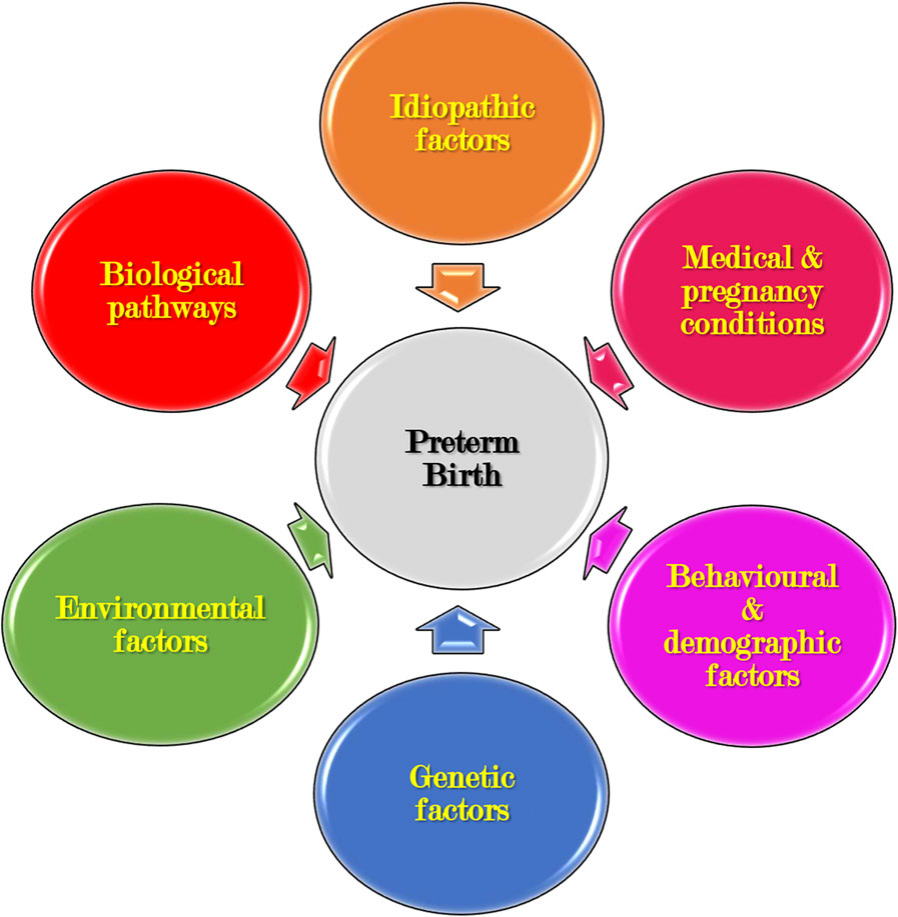
Risk factors associated with preterm birth

Many factors have been attributed to increase the risk of PTB. These factors have been reviewed in several reports previously [8, 9, 12, 13]. The risk factors can be broadly grouped into following major categories with a brief introduction for each category and are illustrated in Fig. 1.

#### Behavioral and sociodemographic factors

This category represents most of the maternal demographic risk factors including socio-economic status, race, age, marital status and educational level. Women belonging to African ethnicity have a higher risk of PTB. Women with low socio-economic status, lower age, lower educational level, or are unmarried have higher risk of PTB. Social behaviors such as smoking, drinking, illicit drug use, and other risky social activities also increase the predilection for PTB. Nutritional deficiencies such as vitamin D, folic acid, and iron deficiency as well as low prenatal body weight or obesity are risk indicators for PTB. Women who suffer from anxiety, depression or have stressful work environment are also at increased risk of PTB. Physical or sexual abuse or inadequate social support also puts pregnant women at the risk of PTB. More information on behavioral and sociodemographic factors is available in recent reviews [8, 9, 12, 13].

#### Medical and pregnancy conditions

Multiple gestations, previous history of PTB, or short inter-pregnancy interval are risk factors for PTB. About 40 % twin pregnancies have gestation length of less than 37 weeks and almost all of the higher multiple gestations end in PTB. A prior history of PTB increase the risk of next delivery by 2.5 folds. Uterine distension or vaginal bleeding are also risk factors for the PTB. Medical disorders such as hypertension, diabetes, and thyroid problems in pregnant women also increase the risk for PTB. Cervical shortening or cervical insufficiency due to trauma or malformations also increase the risk of PTB. Medical and pregnancy conditions predisposing for PTB have recently been reviewed in detail [8, 9, 12, 13].

#### Genetic factors

It has been increasingly evident that genetic factors play a significant role in the etiology of PTB. Preterm birth has tendency for familial aggregation. Women who were born preterm have higher risk of PTB; furthermore, the risk of PTB increases by 80 % in women with sisters who have experienced PTB. Studies on interracial couples have shown that women married to men of African American ethnicity have higher prevalence of PTB. Several studies have been done on the genetic association of single nucleotide polymorphisms (SNPs) in candidate genes to predict their association with PTB. The focus of this review is the exhaustive list of genetic studies that have been reported since 2007 and these will be discussed in detail later in the paper. For details about genetic studies before 2007, several reviews are available [8, 9, 12].

#### Environmental factors

Ever-increasing environmental pollutants and xenobiotics are turning out to be additional important factors for PTB. For example, bisphenol A, a plastic based environmental pollutant has recently emerged as a risk factor for PTB. Air pollution either by itself or through interaction of particulate matter with genetic polymorphisms has also been reported to be a risk for PTB. Sulphur dioxide air pollution is another environmental factor that has been associated with PTB. Living in the vicinity of coal burning power plants in Croatia was reported to increase the incidence of the PTB. Women living in the vicinity of petroleum and petrochemical industries and ship building industries in China also experienced increased incidence of PTB. Several studies have been done on agricultural pesticides and herbicides and some of them have shown to increase the incidence of PTB with others remaining equivocal. With increasing use of computers and computer related products, and the associated accumulation of waste pollutants in the environment, the need is to be on the side of caution as their effects on the outcome of pregnancy remain largely unknown/unpredictable. The association of environmental factors with PTB has been reviewed in detail [9].

#### Biological pathways

Common biological pathways that play important role in the pathogenesis of PTB are infections and inflammation, maternal-fetal hypothalamopituitary adrenal activation, decidual defects, and pathological uterine overdistension. PTB has multifactorial origins and often several systems in the body and many different factor classifications interact in increasing the risk of PTB. Microbial infections are a major risk factor for PTB and this can be as a result of systemic intrauterine or infections or lower genital tract infections. According to some conservative estimates, microbial intrauterine infections account for about 25–40 % of PTBs. The mechanism apparently is through innate immunity activation that leads to interaction of toll-like receptors, inflammatory cytokines, and chemokines. Endotoxins lead to production of prostaglandins which stimulate the uterine contractility resulting in premature labor. Uteroplacental ischemia also leads to PTB. Several studies have indicated that periodontal disease causes PTB possibly due to heightened inflammatory state. More information about biological pathways that predispose to PTB is available in several recent reviews [8, 9, 12, 13].

#### Idiopathic factors

Preterm birth is a multifactorial syndrome and the exact cause of the condition remains poorly defined. It is estimated that only 50 % PTBs can be assigned to a known risk or causative factor, while the rest remain idiopathic.

### Genetic polymorphisms associated with PTB

The initial approach to identify genetic variants conferring an increased risk for PTB was to select candidate genes based on the functional association with a major system in the pathway of PTB. Recently more and more studies are non-candidate driven focusing on genome wide association studies in which thousands of polymorphisms are screened at once. In contrast to earlier methods in which only few genetic regions of specific genes are investigated, the genome wide association studies target the whole genome. Genetic polymorphisms associated with PTB are grouped under the following body systems for descriptive purpose and genes are listed in Additional file 1: Table S1.

#### Endocrine system related gene polymorphisms

Human pregnancy is dependent on the coordinated synthesis, secretion, and interplay of hormones among the fetus, mother, and placenta. Childbirth also involves sequential structural and functional changes in the uterus and cervix effected by coordinated interplay of hormonal mechanisms during the terminal stages of the pregnancy. During parturition uterus changes from a state of quiescence and relaxation during pregnancy to a state of regular, recurring, and synchronous contractions probably owing to removal of mechanisms that promote uterine relaxation [14–16]. The decrease in progesterone concentrations that occurs at the time parturition is one of the examples of loss of quiescence factors at the end of gestation. Decrease in progesterone is simultaneously associated with up-regulation of gene expression for the oxytocin receptor that plays a crucial role in parturition [14, 17]. Progesterone treatment is commonly and effectively used to prevent recurrences of preterm labor [18]. Several studies have been done recently on the association of polymorphisms in the progesterone receptor (*PGR*) gene with PTB. However the results have been equivocal. Ehn et al. [19] analyzed 17 SNPs of *PGR* gene in 415 PTB families and identified significant associations were for both the mother and the preterm baby [19]. Similarly, other studies reported *PGR* gene polymorphisms for minor allele (G for rs471767 or rs1942836) in women with PTB [20, 21]. Furthermore, they were more likely to carry to carry the GT haplotype across rs471767 and rs578029 compared to women with term pregnancy. Progesterone receptor SNPs were also associated with beneficial or harmful treatment-genotype interaction in African American, Hispanic, and Caucasian women who received progesterone caproate treatment for recurrent PTB prevention [22]. Several other studies, however, did not find any association between *PGR* SNPs and PTB [23–25]. No genetic polymorphisms were also found in mitochondrial localization sequence of the truncated progesterone receptor (PR-M) gene in a group of women at high risk for PTB [26].

Oxytocin (*OXT*) and its receptor (*OXTR*) are other crucial hormone system involved in childbirth [27–29]. Oxytocin causes myometrial contractions and helps in assisting cervical ripening [30, 31]. Oxytocin receptor gene is expressed in myometrial and endometrial tissue and also in many other tissues of the body including the central nervous system [28]. To-date many SNPs (several dozen) have been reported in the *OXTR* gene, however, less is known about their association with physiological processes. Recently some studies have reported association of *OXTR* gene polymorphisms with PTB risk [32–34]. An exhaustive study was done in 651 PTB infants and their parents for analyses of 16 single nucleotide polymorphisms (SNPs) in genes for *OXT* and *OXTR*, and leucyl/cystinyl aminopeptidase (*LNPEP*) [35]. Two common SNPs of *OXTR* (rs4686302, rs237902) were shown apparently to be associated with PTB. Kuessel et al. [34] studied four common *OXTR* polymorphisms (rs53576, rs2254298, rs237911, rs2228485) in 100 women each with PTB and term birth. Although no relationship was revealed for each of the four individual polymorphisms and PTB, however, the haplotype combination of rs2228485 C allele, rs2254298 A allele, and rs237911 G allele was found to be significantly associated with an increased risk of PTB.

Relaxin is an important hormone involved in softening of pubic symphysis and collagen remodeling during pregnancy [36]. Serum levels of relaxin are lower in early pregnancy and higher during late pregnancy in women with PTB compared to women with term birth [37]. Increased expression of intrauterine relaxin occurs in women with PPROM [38]. Risk of PTB is high in mothers having homozygosity of the rarer allele (rs10115467 and rs4742076) in the relaxin 2 gene (*RLN2* [39]). In a Filipino population, a SNP in *RLN2* promoter (rs4742076) was associated with the elevated decidual *RLN* expression and PPROM, while as rs3758239 was associated with both PPROM and PTB [40].

Polymorphisms in several other genes related to endocrine system have been reported to increase or decrease the risk of PTB. In Finnish and African American mothers SNPs in follicle stimulating hormone receptor (*FSHR*) gene were associated with PTB [41, 42] and were predicted to likely disrupt zinc finger E-box binding homeobox 1 (*ZEB1*) and elongation factor 3 (*EL*F3) transcription factor binding sites [42]. In Norwegian population, a polymorphism in prostaglandin E receptor 3 gene (*PTGER3*; rs977214) in women exerted a protective effect against PTB [43]. *PTGER3* is a gene associated with inflammatory response and plays a role in initiation of labor. However, another study found an increased risk of PTB in women with SNP in *PTGER3* [44]. Polymorphisms in prostaglandin D receptor (*PTGDR*) also increased the risk of post-coital preterm birth in women [45]. Polymorphism in type 1 insulin-like growth factor receptor (*IGF1R*) in fetus [46] and insulin-like growth factor binding protein 3 (*IGFBP3*) in mother [47] also increased the risk of PTB. Polymorphisms in corticotropin-releasing hormone receptor 2 gene (*CRHR2*) were not associated with increased risk of PTB [48], but a linkage candidate gene approach for identifying genetic variants playing role in PTB found *CRHR1* in infants associated with risk for PTB [47]. The endocrine related genes associated with PTB are listed in Additional file 1: Table S1.

#### Tissue remodeling and biogenesis related gene polymorphisms

Many of the changes in molecular remodeling of the tissues and myometrial contractions during parturition are not yet known or fully characterized. Functional genomic approach has been used during the last decade to analyze gene expression in human [49–51] and rodent myometrium [50, 52]. These studies revealed a number of genes that might be important in the molecular control of initiation of parturition. Comparison of uterine tissue samples of pregnant women in labor and before labor revealed differential expression of genes for myometrial gap junction protein connexin-43 [53], estrogen and progesterone receptors [54], prostaglandin endoperoxide synthase 1 and 2 (*PTGS1* and *PTGS2*) [55], intercellular adhesion molecule-1 (*ICAM1*) [56], 15-hydroxyprostaglandin dehydrogenase (*HPGD*) [57], interleukin-8 (*IL8*) [58], and prostaglandin-endoperoxide synthase 2 (*PTGS2*), and calgranulin B (*S100A9*) [17].

Recent studies have also shown that DNA variants in genes involved in extracellular matrix metabolism such as tissue inhibitor of metalloproteinase 2 (*MMP-2*) and collagen type IV alpha-3 chain (*COL4A3*) were found to increase the risk of PTB [32, 33]. Heat shock protein 47 (*SERPINH1*) which is involved in the maturation of collagen molecules is enriched in African and African American populations and has been found to be associated with PTB [12]. Polymorphism in genes like TIMP metallopeptidase inhibitor 2 (*TIMP2*), *COL1A2*, and endothelin 1 (*EDN1*) almost double the risk of PTB and both maternal and fetal DNA variants are associated with spontaneous PTB [32, 33]. In African American women, an analysis of 1500 SNPs resulted in identification of a susceptibility locus on chromosome 7 which mediates PTB. The locus contains many potential genes including *COL1A2* [22]. In another study [59], polymorphisms in *COL5A2* and *COL5A1* were associated with spontaneous PTB. In contrast, lysyl oxidase-like 1 (*LOXL1*) which is involved in connective tissue biogenesis does not contribute significantly to risk of PTB or PPROM [60]. Similarly, another study [61] also found no evidence of association between spontaneous PTB and polymorphisms in *MMP-1* (1607 1G/2G) and *MMP-9* (1562 C/T) genes. The tissue remodeling and biogenesis related genes associated with PTB are listed in Additional file 1: Table S1.

#### Vascular and angiogenesis related gene polymorphisms

Uteroplacental ischemia or hemorrhage is one of the risk factors of spontaneous PTB and bout 15 % PTBs are due to impairment of placentation [62, 63]. Andraweera et al. [62] studied 1190 nulliparous caucasian women and identified that interactions of polymorphisms in vascular endothelial factor A (*VEGFA*) and angiopoietin 1 (*ANGPT1*) with maternal body mass index (BMI) were associated with higher risk of PTB. Genes regulating renin-angiotensin system have also been implicated in PTB. Angiotensinogen (*AGT*) 174–235 region has been associated with PPROM [64]. Uma et al. [65] demonstrated that polymorphisms in angiotensin converting enzyme gene (*ACE*) in fetal genotype were associated with higher risk of PTB. Several other studies reported that variants of the adrenergic receptor beta 2 gene (*ADRB2*), inducible nitric oxide synthase (*NOS2A*), thrombomodulin, endothelial nitric oxide synthase (NOS3A), plasminogen activator inhibitor-2, and alpha adducin (*ADD1*) were associated with PTB [66, 67]. Coagulation/thrombophilic pathway was predicted to influence the PTB and interestingly, the plasminogen activator inhibitor-1 (*SERPINE1*) polymorphism was found to be associated with PTB [68]. Genetic analysis of 542 women with PTB and 1141 women with term deliveries identified that Factor V (*F5*) gene SNPs interacted with maternal smoking and increased the risk of PTB [69]. In contrast, thrombophilic gene polymorphisms did not haveany association with PTB [70]. Small conductance calcium-activated potassium channel 3 (*KCNN3*) plays a role in smooth muscle relaxation and arterial tone. Polymorphisms in maternal *KCNN3* gene (rs1218585, rs1218584, rs883319) have been found to increase the risk PTB [21, 71]. The vascular and angiogenesis related genes associated with PTB are listed in Additional file 1: Table S1.

#### Metabolism related gene polymorphisms

The cytochrome P450 isoenzymes are involved in the metabolism of organic molecules and the biosynthesis of steroids, lipids, and vitamins. Steroid biosynthetic pathway is an important pathway in the pregnancy maintenance and embryonic development and survival. Source of cholesterol in mother depends on the dietary intake and synthesis within the body whereas that of the fetus is dependent on the mother through placental circulation [72]. Cholesterol is necessary for synthesis of steroid hormones in the mother, placenta and the fetus. Large amounts of maternal steroid hormones are metabolized by the placenta in order to allow normal development of the fetal organs and regulate the pregnancy. During pregnancy the maternal blood lipid levels and mutations in cholesterol metabolism are associated with a risk for PTB [73–75]. Lanosterol 14α-demethylase (*CYP51A1*) is a regulatory enzyme involved in later stage of cholesterol biosynthesis. Analysis of *CYP51A1* polymorphisms in 188 women with PTB and in 188 PTB infants suggested new links between PTB and cholesterol synthesis. Absence of a single allele for *CYP51A1* in a preterm infant/mother combination though with low frequency conferred a risk for PTB [76]. In a separate study, fatty acid metabolism pathway genes were reported to exhibit modest association with PTB [77]. Polymorphisms in several cholesterol metabolism genes such as 7-dehydrocholesterol reductase (*DHCR7*), 3-hydroxy-3-methyl-glutaryl-CoA reductase (*HMGCR*), apolipoprotein A-I (*APOA1*), and ATP-binding cassette transporter (*ABCA*) in the mother and fetus were found to increase the risk of PTB [74]. A linkage candidate gene approach for identifying genetic variants playing role in PTB found cytochrome P450 2E1 (CYP2E1) in infants and ectonucleotide pyrophosphatase /phosphodiesterase family member 1 (*ENPP1*) and *DHCR7* in mothers associated with PTB [47].

Glutathione S-transferase μ 1 (*GSTM1*) enzymes are involved in xenobiotic metabolism that includes detoxification of electrophilic compounds such as environmental toxins, therapeutic drugs, carcinogens, and products of oxidative stress. In Korean women, gene-environment (particulate matter) interaction study between the *GSTM1* null genotype conferred a higher risk of PTB [78]. Lee et al. [79] reported that *GSTM1* null genotype confers risk of PTB independent of serum paraoxonase/arylesterase 1 (*PON1*) genotype in Korean pregnant women. *PON1* causes hydrolysis of the organophosphorus compounds and it was found that a fetus with the susceptible *PON1* (-108TT) genotype was at higher risk of PTB [80]. In 434 Norwegian mother-baby diads, the most significant association with PTB was for a polymorphisms in fetal *PON1* [43]. Interaction of organochlorine pesticides with SNPs in *CYP1A1m2* and *GSTM1* null genotypes may magnify the risk of PTD [81]. An analysis on 1030 African American mothers for African ancestry characters and polymorphisms in CYP1A1 and glutathione S-transferase theta-1 (*GSTT1*) in relation to pregnancy outcome revealed significant contributions of African ancestry and *CYP1A1*- and *GSTT1*-smoking interactions to PTB [82]. Similarly, maternal *CYP1A1* heterozygous mutation and homozygous mutation in combination with *GSTT1* null genotypes confers the risk of PTB [83]. Association of *GSTT1/GSTT2* and P4501A1 with PTB was also reported in other studies [84, 85] suggesting that the combined genotypes of *CYP1A1* and *GSTT1/GSTT2* help identification of pregnant women who are at higher risk of spontaneous PTD owing to passive smoking.

Polymorphisms in C-1-tetrahydrofolate synthase (*MTHFD1*), which is involved in folate metabolism did not show any association with PTB [86, 87]. However, serine hydroxymethyltransferase (*SHMT*) enzyme that plays a role in folate metabolism was associated with PTB [88]. Other recently identified gene polymorphisms associated with PTB include peroxisome proliferator-activated receptor gamma (*PPARG*) [89] and FokI vitamin D receptor (*VDR*) [90]. The metabolism related genes associated with PTB are listed in Additional file 1: Table S1.

#### Innate immunity and inflammation related gene polymorphisms

Immune and inflammatory related gene polymorphisms are by far the most studied polymorphisms in relation to spontaneous PTB. Independent reviews focusing solely on the inflammatory related genetic risk factors targeting years until 2009 are available [91, 92].

Inflammation and immunity processes are a multipronged biological reaction of the body tissues to harmful pathogens and involves cellular and humoral responses. Cytokines, antibacterial proteins, and antibodies synthesized and secreted in the body protect against the infections. Genetic polymorphisms regulating the maternal immunologic response during pregnancy may cause an insufficient or exaggerated inflammatory response to microbial infections and lead to an increased risk of preterm birth. Interleukins are cytokines involved in inflammatory response and IL1 modulates inflammatory responses through integrins on leukocytes and endothelial cells. Polymorphisms in the *IL1α* gene in Japanese women were associated with PTB [93] but *IL1β* polymorphism with or without bacterial vaginosis did not confer any increased risk for PTB [94]. IL1β is a proinflammatory cytokine and an important mediator of innate immune response to microbial products. In a Brazilian population with known high rate of PTB, mothers carrying polymorphisms in intron 2 of IL1 receptor antagonist (*IL1RN*) gene were found to have increased susceptibility to PTB [95]. IL1 receptor antagonist binds to IL1 receptor 1 (*IL1R1*) and inhibits the association of IL1R1with the coreceptor for signaling thus blocking the IL1 activity. Also, maternal carriers of *IL1RN* polymorphism in a group of Polish women were at increased risk of PTB and this risk was further elevated with a coincidence of at least one copy of *IL6* allele G [96]. In non-Hispanic white women, higher risk of PTB was found when both mother and child carried the *IL1RN* intron2 repeat [97]. Interleukin 6 is a proinflammtory cytokine released by T-cells and macrophages and stimulates inflammatory response during infection. Studies on European Americans and African Americans found potential role of polymorphisms of *IL6* and IL6 receptor (*IL6R*) genes in PTB and in the differential disparity for the risk in the two ethnicities [98]. Genetic variations in *IL6* and *IL6R* were found to be associated with increased amniotic concentration of IL6 in PTB patients [99].

Periodontitis was suggested to be associated with PTB but a study on Japanese women did not find any association between PTB and periodontitis [100]. However, polymorphisms in Fc fragment of IgA receptor (*FCAR*) and *IL6* increased the risk for PTB in the same study. In contrast, other studies [101, 102] have found no association of polymorphism in *IL6* and *IL10* with PTB. Interleukin 10 is an anti-inflammatory cytokine and attenuates the inflammatory response through effects on proinflammatory cytokines and reduces the function of host immune cells, such as neutrophils and macrophages. Polymorphism in genes for Protein kinase C alpha (*PRKCα*) and *IL6* interact with bacterial vaginosis and confer increased risk for PTB [103]. An exhaustive study on 402 PTB mother-fetus combinations and 1227 term mother-fetus combinations for analysis of 775 SNPs in 190 candidate genes found that *IL6R1*, involved in controlling fetal inflammation, doubled the risk for PTB [33]. In contrast, maternal carrier of polymorphic alleles of *IL1β* (+3953C > T), *IL6* (-174G > C), and *IL1RN* genes did not show any association with PTB due to PPROM [104]. In African American population, an ethnic group with a known higher rate of PTB, polymorphisms in infection and inflammation related genes particularly *IL12* and IL12 receptor beta (*IL12Rβ*) were found to confer elevated risk for PTB [105]. Similarly, haplotypes of *IL13/IL4* in a German population were also associated with PTB [106].

Tumor necrosis factor (TNF) α is secreted by immunocompetent cells after contact with microbial metabolites and mediates the immunologic response of the host. TNFα shows significant variability among individuals possibly due to the genetic polymorphisms. Studies have shown that polymorphisms in *TNF*α promoter confer an increased risk of PTB [94, 107]. In Chinese Han population polymorphisms in TNF receptor (*TNFR2*) gene did not increase the risk of PTB but 96 TG (GG) genotype may contribute to susceptibility to chorioamnionitis in the process of PTB [108]. The increased risk of PTB in Danish Caucasian women in association with polymorphisms of *TNFα* and *IL1β* was attributed to dysregulation of the immune system in pregnancy [109]. Moura et al. [92] reported that polymorphisms in *TNFα*, interferon (*IFN*)γ, and *IL6* were associated with elevated risk of PTB. *TNFα* gene polymorphism G308A was associated with PTB and the risk of PTB was reduced if mother's or child's genotype was G/A [110]. In several other studies *TNFα* gene polymorphisms were consistently associated with PTB [111–113]. Among African-American women, chances of PTB were found to be higher if both mother and child carried the *TNFR2 M196R* allele [97]. Polymorphisms in *TNFα* also interacted with HEV infection during pregnancy and increased the risk of PTB [114]. In contrast, other studies did not find any association of *TNFα* -G308A mutation with the risk of PTB [115] or PTB due to PPROM [104]. A linkage candidate gene approach for identifying genetic variants playing role in PTB found TNF receptor-associated factor 2 (*TRAF2*) gene variants in mothers associated with PTB [47].

Toll-like receptors (*TLRs*) are proteins involved in innate immune response and are expressed in macrophages and dendritic cells. Toll-like receptors interact with invading microbes and activate immune cell response triggering production of cytokines and chemokines. Polymorphisms in *TLR* 2 variant confers increased risk of PTB [116]. Similarly, fetuses that carry the Asp299Gly substitution in *TLR4* conferred an increased risk of PTB [117]. A study in German population suggested an elevated risk of PTB with haplotypes of *IL4, IL13*, and *TLR10* [106]. In contrast, maternal carriage of *TLR4 1196 T* allele may be associated with reduced risk of PTB before 33rd week of gestation in Polish population [118]. Polymorphisms in *TLR* pathway adapter variant *TIR* domain receptor-associated protein (*TIRAP*) have been found to render protection against PTB [119].

Polymorphisms in *ICAM1* have also been found to confer risk for PTB in a Korean population [120]. *ICAM1* has a proinflammatory role in facilitating leukocyte endothelial transmigration by signaling through cascades involving a number of kinases. Polymorphisms in low affinity immunoglobulin gamma Fc region receptor II-b (*FCGR2B*) gene which is involved in regulation of antibody production and apoptosis also confer a higher risk for PTB [121]. Gene-environment interactions of polymorphisms for protein kinase C alpha (*PRKCα*), fms-like tyrosine kinase (*FLT1*), and IL6 with bacterial vaginosis contributed to the risk of PTB [103].

Several other studies recently have reported the association of polymorphisms for many other immune and inflammatory genes with PTB. These studies include the genes such as defensin α 5 (*DEFA5*) [32, 33], *IL1β* [122], major histocompatibility complex, class II, DQ alpha 1 (*HLA-DQA1*) [123], compliment region 1 (*CR1*) [124], selenoprotein (*SEPS1*) [125], surfactant pulmonary-associated protein D (*SFTPD*) [126], colony-stimulating factor 2 (*CSF2*) [127], interferon γ receptor 2 (*IFNGR2*) [127], and killer cell immunoglobulin-like receptor three domain long cytoplasmic tail 2 (*KIR3DL2*) [127]. The full list of the genes that have been reported with a potential association with PTB is presented in Additional file 1: Table S1.

#### Miscellaneous gene polymorphisms

Miscellaneous section contains information on genetic polymorphisms that could not be included in any other section and the number of studies was too small to create separate sections for them. Analysis of nonsynonymous mitochondrial DNA (*mtDNA*) variants A4917G, G10398A and T4216C for potential interaction with smoking and association with PTB was done in 422 pregnant Caucasian women [99]. Mitochondrial DNA variants A4917G and T4216C conferred risk for PTB after adjusting for smoking, indicating that mitochondrial genome polymorphisms may play a significant role in PTB through an interaction with smoking. In contrast, no mitochondrial gene variant was found to contribute to the maternal transmission of PTD in another study [128]. In an African American population, polymorphisms in fetal catechol-o-methyltransferase (*COMT*) gene (rs4818) conferred increased risk of PTB [129]. Several signaling factors such as early growth response 1 (*EGR1*), transcription factor AP2A (*TFAP2A*), and specificity protein 3 (*SP3*) have been suggested as useful in PTB risk prediction [130]. X-chromosomal SNPs data on a Norwegian, Danish and Argentinean populations showed that the G allele of maternal SNP rs2747022 in the FERM domain containing 7 (*FRMD7*) gene conferred increased risk for PTB [131].

## Conclusions

Preterm birth is increasingly being recognized as a complex syndrome for which the etiology is not yet fully understood. Lack of a well-defined etiology is a major limitation in predicting impending spontaneous preterm birth and instituting effective preventive measures. Therefore, the challenge is to identify high risk women and provide them a personalized medical care for reducing the burden of PTB. In addition to infectious, lifestyle, and demographic risks, genetic factors increasingly seem to be important in PTB. Familial aggregation of PTB and recurrent occurrence in women puts forth a case of complex genetic predisposition. Study of genetic polymorphisms can be useful in predicting the risk for PTB. It has been estimated that SNPs constitute about 90 % of all genetic variations in the human genome. During the last decade studies on functional candidate gene variants and genome-wide linkage analyses in women and families undergoing recurrent preterm birth have thrown forward a large number of potential genes predisposing for preterm birth. Majority of the significant associations for SNPs and PTB are related to inflammation. Many of the polymorphisms exhibit inconsistency and remain inconclusive. Being a global problem preterm birth warrants global solutions. Recent genomic approaches are beginning to reveal the information for understanding the causes of PTB. However, to fully appreciate and understand the complexity of PTB, future approaches using high-throughput genome sequencing methods such as whole-exome sequencing and whole-genome sequencing studies are essential. These may involve multicenter national and international collaborative efforts and may also include proteomic and metabolomics approaches that will significantly enhance the understanding of PTB ultimately leading to the formulation of preventive interventions.

## Additional file

Additional file 1: Table S1. Single nucleotide polymorphisms associated with a risk for preterm birth. (DOCX 17 kb)

## Abbreviations

IOM: Institute of medicine
PTB: Preterm birth
SNPs: Single nucleotide polymorphisms
WHO: World health organization
