# Supplementary material for: Spontaneous preterm birth and single nucleotide gene polymorphisms: a recent update
Source: BMC Genomics. 2016 Oct 17;17(Suppl 9):759. doi: 10.1186/s12864-016-3089-0 (PMC5073925; doi:10.1186/s12864-016-3089-0)
Supplement: Additional file 1: Table S1. — Single nucleotide polymorphisms associated with a risk for preterm birth. (DOCX 17 kb) [file 12864_2016_3089_MOESM1_ESM.docx]

**Additional file: Table S1. List of genes that have been studied for single nucleotide polymorphisms associated with a risk for preterm birth.**

| **Systems** | **Name of the gene (nomenclature) [reference]** |
| --- | --- |
| **Endocrine system related genes** | Corticotropin receptor 1 *(CRHR1)* [47]  Follicle stimulating hormone receptor (*FSHR*) [41,42]  Glucocorticoid receptor *(NR3C1)* [47]  Insulin-like growth factor 2 (*IGF2*) [32,33]  Insulin-like growth factor receptor 1(*IGF1R)* [46,47]  Leucyl/cystinyl aminopeptidase (*LNPEP*) [35]  Oxytocin (*OXT*) [35]  Oxytocin receptor *(OXTR)* [34,35,47]  Progesterone receptor (*PGR*) [19–22, 24, 25, 47]  Prostaglandin E receptor 3 (*PTGR3*) [43,44]  Prostaglandin E synthase 2 (*PTGES2)* [77]  Prostaglandin G/H synthase 1 *(PTGS1)* [47]  Prostanoid DP receptor (*PTGDR*) [45]  Relaxin 2 gene (*RLN2*) [39,40] |
| **Tissue remodeling and biogenesis related genes** | Collagen type I (*COL1A2*) [22,32,33,43,59]  Collagen type IV (*COL4A2*) [32,33]  Collagen type IV (*COL4A3*) [32,33]  Collagen type IV (*COL4A4*) [32,33]  Collagen type IV (*COL4A5*) [32,33]  Collagen type IV (*COL4A6*) [32,33]  Collagen type V (*COL5A2*) alpha-2 [59]  Intercellular adhesion molecule-1 (*ICAM1*) [120]  Matrix metalloproteinase 1 (*MMP-1*) [61]  Matrix metalloproteinase 8 (*MMP-8*) [43]  Matrix metalloproteinase 9 (*MMP-9*) [61,97]  Matrix metalloproteinase 10 (*MMP-10*) [32,33]  Matrix metalloproteinase 16 (*MMP-16*) [32,33]  Tenascin-R (*TNR*) [32, 33]  TIMP metallopeptidase inhibitor 2 (*TIMP2*) [32,33] |
| **Vascular and angiogenesis related genes** | Alpha adducin (*ADD1*) [66]  Angiopoietin 1 (*ANGPT1*) [62]  Angiotensin converting enzyme (*ACE*) [64,65]  Angiotensin II receptor type 1 (*AT1*) [64]  Angiotensinogen (*AGT*) [64, 86]  Beta-2 adrenergic receptor (*ADBR2*) [66,67]  Complement receptor 1 (*CR1*) [124]  Cyclin-dependent kinase 4 inhibitor (*CDKN2*) [123]  Endothelial nitric oxide synthases (*NOS3*) [66,67]  Endothelin 1 (*EDN1*) [32,33]  Factor V (*F5*) [69,86]  Inducible nitric oxide synthases (*NOS2*) [66,67]  kinase insert domain receptor (*KDR*) [62]  Peroxisome proliferator-activated receptor gamma (*PPARG*) [89]  Plasminogen activator inhibitor-1 (*SERPINE1)* [66,68]  Renin (*REN*) [64]  Small conductance calcium-activated potassium channel 3 (KCNN3) [21,47,71]  Thrombomodulin (*THBD*) [66]  Vascular endothelial growth factor (*VEGFA*) [62] |
| **Metabolism related genes** | Apolipoprotein A-I (*APOA1)* [74]  Apolipoprotein C (*APOC)* [74]  Apolipoprotein E (*APOE*) [74]  ATP-binding cassette transporter (*ABCA1)* [74]  Cholesteryl ester transfer protein (*CETP)* [74]  Cytochrome P4501A1 (*CYP1A1*) [76,78,81–85]  Dehydrocholesterol reductase (*DHCR24)* [74]  FC alpha Receptor (*FCαR*) [100]  Glutathione S-transferase mu 1 (*GSTM1*) [78,79,81,84]  Glutathione S-transferase theta 1 (*GSTT1*) [78,82–85]  Glutathione S-transferase theta 2 (*GSTT2* ) [85]  Glutathione S-transferase theta pseudogene (*GSTTP1* ) [85]  Hepatic lipase (*LIPC)* [74]  Hydroxy methyl glutaryl CoA reductase (*HMGCR*) [74]  Lipoprotein lipase (*LPL*) [123]  Mannose binding lactin (*MBL*)[132]  Methionine synthase (*MTR*) [86]  Methionine synthase reductase (*MTRR*) [86]  Methylene tetrahydrofolate reductase (*MTHFR*) [86,88]  Methylenetetrahydrofolate dehydrogenase 1 (*MTHFD1*) [87]  Serine hydroxymethyltransferase 1(*SHMT1*) [86]  Serum paraoxonase/arylesterase 1 (*PON1*) [43,59,80]  Vitamin D receptor *(VDR)* [47] |
| **Innate immunity and inflammation related genes** | Colony-stimulating factor 2 (*CSF2*) [127]  Defensin alpha 5 (*DEFA5*) [32, 33]  Fms-like tyrosine kinase 1 (*FLT1*) [133]  HLA class II histocompatibility antigen, DR alpha chain (*HLA-DRA*) [123]  HLA class II histocompatibility antigen, DRB1-9 beta chain (*DRB1*) [123]  Interferon γ (*IFN-γ*) [92,114]  Interferon γ receptor 2 (*IFNGR2*) [127]  Interleukin 1 alpha (*IL1α***)** [43,93,112]  Interleukin 1 beta (*IL1β*) [94,109,112,122]  Interleukin 1 receptor 2 (*IL1R2*) [43]  Interleukin-1 receptor antagonist *(IL1RN)* [95–97]  Interleukin-1 receptor-associated kinase 1 *(IRAK1)* [119]  Interleukin 2 (*IL2*) [32,33]  Interleukin 2 receptor beta (*IL2Rβ*) [105]  Interleukin 4 (*IL4*) [43,106,126]  Interleukin 6 (*IL6*) [92,96,98,114,133]  Interleukin 6 receptor (*IL6R*) [32,33,43,98]  Interleukin 10 *(IL10*) [102]  Interleukin 12 *(IL12*) [106]  Interleukin 12 receptor (*IL12Rβ*) [105]  Interleukin 12 alpha (*IL12α*) [126]  Interleukin 13 (*IL13*) [106,126]  Interleukin 15 *(IL15*) [105]  Interleukin 23 receptor (*IL23R*) [123]  Killer cell immunoglobulin-like receptor three domain long cytoplasmic tail 2 (*KIR3DL2*) [127]  Lactotransferrin (*LTF*) [32,33]  low-affinity receptor for immunoglobulin G (*FcγRIIb*) [121]  Major histocompatibility complex, class II (*HLA-DQA1*) [123]  Nuclear factor-kappa B1 (*NFκB1*) [119]  Protein kinase C alpha (*PRKCA*) [133]  Selenoprotein S (*SEPS1*) [125]  Surfactant, pulmonary-associated protein D (*SFTPD)* [126]  TIR domain receptor-associated protein (*TIRAP*) [119]  Tumor necrosis factor alpha (TNF α) [92,94,97,107,109–113,115]  Tumor necrosis factor receptor 2 (*TNFR2*) [97,108]  TNF receptor associated factor 2 *(TRAF2)* [47]  Toll-like receptor 2 *(TLR2)* [116,119]  Toll like receptor 4 (*TLR4*) [117–119]  Toll like receptor 5 (*TLR5*) [119]  Toll like receptor 9 (*TLR9*) [119]  Toll-like receptor 10 (*TLR10*) [106]  Transforming growth factor beta1 (*TGF-β1*) [114] |
| **Miscellaneous genes** | Catechol-o-methyltransferase (*COMT*) [129]  Early growth response 1 (*EGR1*) [130]  FERM domain containing protein 7 (*FRMD7*) [134]  Mitochondrial genome variants [99]  Transcription factor AP2A (*TFAP2A*) [130]  Specificity protein 3 (*SP3*) [130] |
